# New-old hemoglobin-like proteins of symbiotic dinoflagellates

**DOI:** 10.1002/ece3.498

**Published:** 2013-02-26

**Authors:** Nedeljka N Rosic, William Leggat, Paulina Kaniewska, Sophie Dove, Ove Hoegh-Guldberg

**Affiliations:** 1 School of Biological Sciences, The University of QueenslandSt. Lucia, Qld, 4072, Australia; 2 School of Pharmacy and Molecular Sciences and ARC Centre of Excellence for Coral Reef Studies, James Cook UniversityTownsville, Qld, 4811, Australia; 3 Australian Institute of Marine SciencePMB 3, Townsville, Qld, 4810, Australia; 4 Global Change Institute and ARC Centre of Excellence for Coral Reef Studies, The University of QueenslandSt. Lucia, Qld, 4072, Australia

**Keywords:** Coral, functional genomics, Hemoglobin, stress, *Symbiodinium*, Symbiosis

## Abstract

Symbiotic dinoflagellates are unicellular photosynthetic algae that live in mutualistic symbioses with many marine organisms. Within the transcriptome of coral endosymbionts *Symbiodinium* sp. (type C3), we discovered the sequences of two novel and highly polymorphic hemoglobin-like genes and proposed their 3D protein structures. At the protein level, four isoforms shared between 87 and 97% sequence identity for Hb-1 and 78–99% for Hb-2, whereas between Hb-1 and Hb-2 proteins, only 15–21% sequence homology has been preserved. Phylogenetic analyses of the dinoflagellate encoding Hb sequences have revealed a separate evolutionary origin of the discovered globin genes and indicated the possibility of horizontal gene transfer. Transcriptional regulation of the *Hb*-like genes was studied in the reef-building coral *Acropora aspera* exposed to elevated temperatures (6–7°C above average sea temperature) over a 24-h period and a 72-h period, as well as to nutrient stress. Exposure to elevated temperatures resulted in an increased *Hb-*1 gene expression of 31% after 72 h only, whereas transcript abundance of the *Hb*-2 gene was enhanced by up to 59% by both 1-day and 3-day thermal stress conditions. Nutrient stress also increased gene expression of *Hb*-2 gene by 70%. Our findings describe the differential expression patterns of two novel *Hb* genes from symbiotic dinoflagellates and their polymorphic nature. Furthermore, the inducible nature of *Hb*-2 gene by both thermal and nutrient stressors indicates a prospective role of this form of hemoglobin in the initial coral–algal responses to changes in environmental conditions. This novel hemoglobin has potential use as a stress biomarker.

## Introduction

Globin proteins are a diverse group of proteins, organized in a number of families and represented in all kingdoms of life (Vinogradov et al. 2006). Hemoglobin (Hb) proteins are a member of the globin family that contain a prosthetic group (heme) with iron (Fe^+2^) coordinated with the absolutely conserved proximal histidine (Vuletich and Lecomte 2006). The average size of Hb is 140–180 aa (Mr 15–18 kDa) and is characterized by a low homology between distant relatives (Suzuki and Imai 1998). A variety of hemo-proteins that exist in living organisms share similar tertiary structure (globin fold) and evolutionary origin, while displaying a large sequence diversity in their primary structure (Royer et al. 2005). In vertebrates, there are four types of globin proteins including hemoglobin, myoglobin, neuroglobin, and cytoglobin (Pesce et al. 2002). In plants, hemoglobins are divided into symbiotic and non-symbiotic hemoglobins, as well as truncated hemoglobins (Shimoda et al. 2005). Three groups of hemoglobins have been characterized in microorganisms: truncated hemoglobins containing 110–140 aa, flavohemoglobins containing hemoglobin and a flavin-containing reductase domain and myoglobin-like proteins (Egawa and Yeh 2005), with a number of microbial Hbs lacking a completely conserved goblin fold (Bonamore and Boffi 2008). Truncated hemoglobins are also found in bacteria, unicellular eukaryotes, and higher plants (Milani et al. 2005). Furthermore, hemoglobin proteins show a high diversity of their structural and also functional properties. In vertebrates, globin proteins are involved in the capture, transport, and storage of O_2_ and CO_2_, whereas in invertebrates, they have preserved the function of O_2_ binding (Lecomte et al. 2005). From an evolutionary point of view, the oxygen transport function is proposed to be related to the appearance of multicellular animals (Vinogradov et al. 2006). Hbs are also involved in scavenging nitric oxide (NO) and the protection of cells from NO damage (Egawa and Yeh 2005; Lecomte et al. 2005). During severe hypoxia stress in *Arabidopsis*, alfalfa, and maize, over-expressed non-symbiotic plant class 1 hemoglobin has been involved in reducing NO level and increasing overall the plants’ survival rate (Dordas et al. 2003a, 2004; Perazzolli et al. 2004). The importance of Hb in symbiosis has been suggested as high mRNA expression levels of non-symbiotic and truncated Hbs are observed in root nodules of *Lotus japonicus* compared with other plant tissues (Bustos-Sanmamed et al. 2011). Leghemoglobins, symbiotic plant Hbs, which are also found in root nodules of legumes are involved in O_2_ transport to the nitrogen fixing bacteria and are as well required for symbiosis (Ott et al. 2005). Highly polymorphic and diverse Hb sequences indicate their capacity for a potential molecular mechanism of adaptation, and therefore they have been proposed to present a unique system for studying the effect of environmental changes on molecular evolution (Andersen et al. 2009).

**Figure 1:**
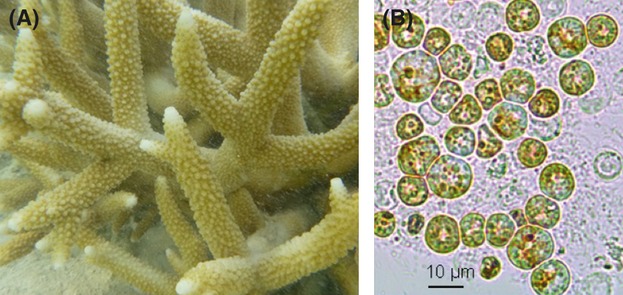
Coral *Acropora aspera* on the Great Barrier Reef, Australia (A). Light micrograph of *Symbiodinium* maintained in culture at constant temperature and light conditions (B).

*Symbiodinium* are unicellular photosynthetic dinoflagellates, involved in a mutualistic symbiosis with a number of marine organisms such as scleractinian corals (Fig. 1; Muscatine et al. 1975; Trench 1979). Symbiotic dinoflagellates are phylogentically separated into nine clades (A–I) and then additionally into multiple subclades (Santos et al. 2002; Coffroth and Santos 2005; Pochon et al. 2006; Pochon and Gates 2010). It has been shown that different *Symbiodinium* clades and subclades can influence the physiological tolerance of the coral–dinoflagellate symbiosis to environmental stress (Rowan 2004; Berkelmans and van Oppen 2006; Robison and Warner 2006; Loram et al. 2007; Reynolds et al. 2008; Sampayo et al. 2008; DeSalvo et al. 2010; Fisher et al. 2012). Recent studies of gene expression levels in symbiotic dinoflagellates following the exposure of the coral–algal symbiosis to elevated temperatures have revealed differential regulation of molecular chaperones (*Hsp*70 and *Hsp*90) and cytochrome P450 genes (Rosic et al. 2010, 2011a,b). Differential responses of *Hsp*70 and *Hsp*90 orthologs from both partners in symbiosis have been also observed under thermal stress conditions (Leggat et al. 2011).

Temperature variation has been shown to affect the demand and the supply of oxygen, suggesting an adaptive role of different hemoglobin isoforms in marine fishes, in optimizing oxygen transport and the levels of oxygen and carbon dioxide under various thermal conditions (Sartoris et al. 2003; Pörtner and Knust 2007). Hbs are also involved in the scavenging of NO, which is a free radical and a membrane-permeable molecule involved in the immune responses, and in establishing and maintaining coral–algal symbiosis (Gardner et al. 1998; Trapido-Rosenthal et al. 2001). The connection between NO and thermal stress has been suggested as elevated temperatures resulted in an increase in NO production in the sea anemone *Aiptasia* followed by a breakdown of the symbiosis (Trapido-Rosenthal et al. 2001; Perez and Weis 2006).

In the present study, we characterize the sequence polymorphisms of two putative hemoglobin genes identified within expressed sequence tags (ESTs) of *Symbiodinium* (clade C3) (Leggat et al. 2007), provide their phylogenetic analyses, and propose the 3D protein structures. In addition, we apply transcriptional analyses to coral dinoflagellates both in symbiosis and in vitro cultures to determine the changes in the gene expression patterns of hemoglobin-like proteins when exposed to different thermal and nutrient stress conditions. Finally, we discuss the potential importance of *Hb* genetic polymorphisms as a tool of evolutionary adaptation.

## Materials and Methods

### Sequence identity and phylogenetic analysis

**Figure 2:**
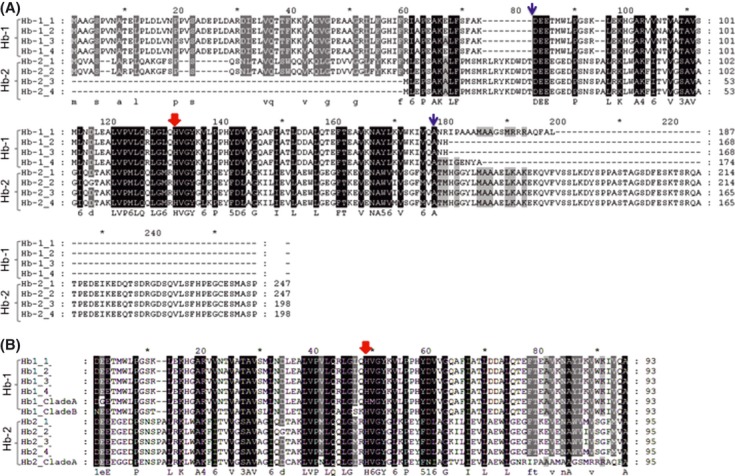
Multiple sequence alignment of the *Symbiodinium* hemoglobin encoding sequences from coral dinoflagellates ESTs (A) including the sequences obtained from different *Symbiodinium* cultures (ITS2-A2 and B2; B) was performed with Clustal W. Predicted amino acid sequences of *Symbiodinium* ESTs designated as *Hb*-1 and *Hb*-2 and their isoforms were aligned to the selected sequences producing the best hits according to blast search (http://blast.ncbi.nlm.nih.gov/Blast.cgi). Within the alignment, identical residues are marked as conserved amino acids that are shaded in black (100% conserved). Residues that are gray shaded with white letters have more than 80% conserved regions or if gray shaded with black letter more than 60% conserved regions. Sequences used in phylogenetic analyses for building a tree are indicated between blue arrows, whereas red arrow indicates the absolutely conserved proximal histidine (F8) from the globin domain.

The sequences of putative hemoglobin genes (Fig. 2) were identified from an EST library of *Symbiodinium* (genotype C3) isolated from the coral host *Acropora aspera* exposed to different stress factors including elevated temperature and a range of ammonium and inorganic carbon concentrations (Leggat et al. 2007). The EST sequences were extracted from the library and identified using BLAST (http://blast.ncbi.nlm.nih.gov/Blast.cgi), search option tblastx. Hemoglobin sequences were aligned using SeqMan software (Lasergene® sequence analysis software, USA). A set of forward and reverse primers were constructed (Table S1) and used to amplify the full length of the hemoglobin open reading frame (ORF).

For initial amplification of the cDNA library, the PCR reaction (30 μL) was done using 3 μL of DNA template (from 1/100 diluted cDNA library), 0.25 μmol/L each of M13 forward and M13 reverse primers, 0.2 mmol/L dNTPs, 2.2 mmol/L MgCl_2_, 1.65U Platinum *Taq* polymerase in buffer (20 mmol/L Tris HCl buffer pH 8.4 containing 50 mmol/L KCl). PCR conditions were as follows: 1 min at 94°C, followed by 35 cycles of denaturation at 94°C for 20 sec, 56°C for 20 sec, and 72°C for 1.5 min. A final extension step at 72°C for 10 min was done prior to storage of reactions at 4°C.

Following PCR amplification using flanking primers generated within the ORFs of hemoglobin, genes was carried out using 2 μL of 1/10 diluted PCR reaction mixture of multiplied cDNA library, 0.25 μmol/L each of forward and reverse primers (Table S1), 0.2 mmol/L dNTPs, 2.2 mmol/L MgCl_2_, 1.65U Platinum *Taq* polymerase in buffer (20 mmol/L Tris HCl buffer pH 8.4 containing 50 mmol/L KCl). The conditions for the gradient PCR were as follows: 1 min at 94°C, followed by 35 cycles of: denaturation at 94°C for 20 sec, annealing in the range of different temperature from 53°C to 66°C for 20 sec and 72°C for 2 min. A final extension stage at 72°C for 10 min was done prior to storage of reactions at 4°C. Bands of an approximate size of 650 bp and 850 bp corresponding to putative *Hb*-1 and *Hb*-2 genes, respectively, were sub-cloned into a pGEM vector following the manufacturer recommendation (pGEM-T Easy, Promega, Australia) and introduced into Top-10 cells (Invitrogen, Australia). Selected colonies were used for plasmid DNA extraction (Qiagen, Australia) and subjected to automated sequencing at the Australian Genome Research Facility (University of Queensland, Australia).

**Figure 3:**
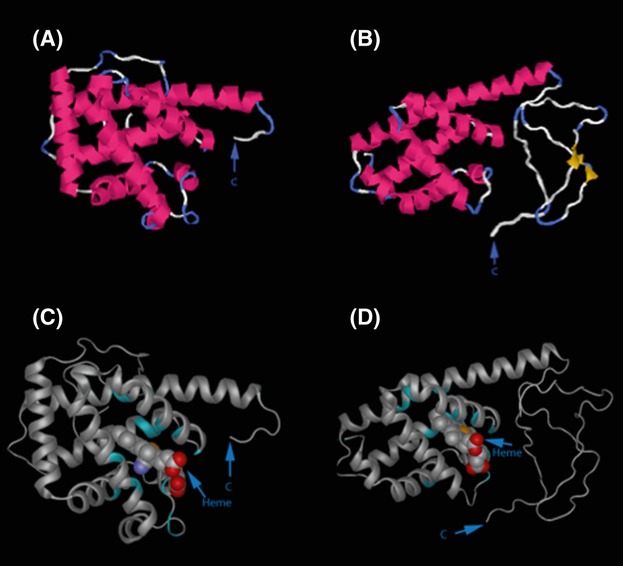
The predicted 3D protein structures of Hb-1 (A) and Hb-2 (B). The letters C correspond to the carboxyl terminals. The predicted binding site residues are indicated in green, including the position of heme ligand in the 3D structural model of Hb-1 (C) and Hb-2 (D).

The representative sequences of analyzed hemoglobin genes were subjected to BLAST search and their accession numbers are shown in Table 1. Sequence analyses were performed using BLAST search options tblastx and blastx, whereas protein sequences were analyzed using tblastn and blastp. The highest scoring hits (E value <10^−5^) were used for further sequence analyses and the generation of a phylogenetic tree. Sequence analysis was performed using web-based BioManager – ANGIS services (http://bioman5.angis.org.au). The multiple sequence alignments of selected sequences were done in ClustalW (Thompson et al. 1994), whereas manual adjustment of alignments was done using GeneDoc software (Nicholas et al. 1997). The prediction of transmembrane areas within the hemoglobin sequences was performed using the multiple sequence alignment and program tmap (Persson and Argos 1994), whereas a search for chloroplast transit peptides was done using web-based TargetP 1.1 Server: http://www.cbs.dtu.dk/services/TargetP/ (Emanuelsson et al. 2007). The secondary and tertiary structures of *Symbiodinium* Hb-like protein sequences were analyzed using the on-line sever I-TASSER (Fig. 3; Zhang 2008; Roy et al. 2010). The predictions of protein structure and function were automatically done using this server and high-quality 3D structures have been obtained.

**Table 1:** GenBank accession numbers, designations, functions, and best BLAST (blastx) hits (E < 1.0 × 10^−5^) for putative *Symbiodinium Hb* genes

Gene name	GeneBank Accession Number	Annotation	Species with the closest similarity(GeneBank accession number)	E value
*Hb-1*	EH035884	Hemoglobin	*Sorangium cellulosum*(YP_001611205)	1.00E -15
		Putative hemoprotein	*Azorhizobium caulinodans* (YP_001523599)	5.00E -15
		Probable bacterial hemoglobin	*Maritimibacter alkaliphilus*(ZP_01012339)	1.00E-13
		Putative nitric oxide dioxygenase (NOD); flavohemoprotein	*Bradyrhizobium* sp.(YP_001204533)	3.00E-12
		Non-vascular plant hemoglobin	*Marchantia polymorpha*(AAK07743)	2.00E-11
*Hb-2*	EH038142	Hemoglobin	*Sorangium cellulosum*(YP_001611205)	1.00E -14
		Putative hemoglobin	*Shewanella amazonensis(*YP_929350)	1.00E-13
		Putative nitric oxide dioxygenase (NOD)	*Bradyrhizobium* sp.(YP_001238856)	2.00E-09
		Cytoglobin	*Spalax judaei(*CAL91964)	2.00E-07
		Cytoglobin	*Homo sapiens*(AAH29798)	2.00E-06

The multiple sequence alignments used in the phylogenetic analysis (Leignel et al. 2007) contained only the conserved region, excluding the variable N- and C-terminal regions. Sequences used in phylogenetic analyses for building a tree are indicated in Fig. 2A. Phylogenetic trees were reconstructed using maximum likelihood estimates based on the Dayhoff PAM matrix. The robustness of phylogenetic tree was assessed using 500 bootstrap replicates (Felsenstein 1989).

### Experimental design

Coral fragments (7 cm long) of *Acropora aspera* (five colonies) harboring *Symbiodinium* C3 genotype (LaJeunesse et al. 2003) were collected from the reef flat at Heron Island, Great Barrier Reef, Australia (23°25′S; 152°07′E) during the Australian winter in June 2009. After collection, the coral fragments were immediately transferred to flow through aquaria, fixed to racks and allowed to acclimatize on the reef flat at approximately 23–24°C (mean ambient temperature during winter at Heron Island) for 2 weeks. To examine the effect of elevated temperatures on *Hb* gene expression in *Symbiodinium*, coral branches were randomly distributed across aquaria (three aquaria per treatment) and then exposed to two experimental temperature regimes: 1-day thermal stress (∼1°C increase per h, 24–32 ± 1°C), 3-day (∼0.5°C increase per h, 23–24 ± 1°C), and a control group (stable at 23–24 ± 1°C; ambient temperature) over a 24-h period for the 1-day heat stress and a 72-h period for the control and medium temperature regimes. Nutrient stress was prepared in a 15-L tank spiked three times (once a day) with 1 mol/L NH_4_Cl reaching a concentration of 20 μmol/L after 3 days. This concentration represents a nutrient stress condition corresponding to 10–20 times greater ammonium concentration than found in nature (Koop et al. 2001; Grover et al. 2002).

For each replicate, aquarium water temperature was measured every 2 min using StowAway TidbiT Loggers (Onset Computer Corporation, Bourne, MA, USA). The maximal temperature applied here was 30°C, which is approximately 6–7°C higher than mean seawater temperature during the course of the experiment. Coral branches (*n* = 5) were sampled from the treatment and control aquaria at T0, T24, and T72 h, immediately snap-frozen in liquid nitrogen and stored at −80°C prior to processing.

### Culturing conditions

Cultures of *Symbiodinium* sp. were obtained from Professor Roberto Iglesias-Prieto (RSU Puerto Morelos, UNAM, Mexico) and identified as clade C (ITS [Internal Transcribed Spacer] – type C1), clades A (ITS type A2), and B (B2). Axenic cultures were grown in f/2 medium (Guillard and Ryther 1962). The maintenance of cultured *Symbiodinium* was done at 25°C, 12:12-h day–night period, with an irradiance of ∼40 μmol quanta/s^1/^m^2^ (measured using a Li-Cor flat quantum sensor). The algal cells were centrifuged and the resulting pellet was snap-frozen in liquid nitrogen and stored at −80°C prior to RNA extraction.

### Total RNA extraction

RNA was extracted from coral branches using a small fragment (0.5–1 cm long) that was cut with a bone cutter and crushed directly in liquid nitrogen. The obtained powder was put in Trizol, homogenized with a hand homogenizer (Tissue-Tearor, Biospec products, Inc.) and centrifuged for 3 min at 13,000 × *g* at 4°C. The aqueous phase was then used for the extraction of total RNA with RNeasy kit (Invitrogen, Australia) following the manufacturer's instructions. The RNA quantity and integrity was analyzed on an Agilent 2100 Bioanalyzer and 500 ng of high-quality total RNA (integrity number > 7). In addition, RNA isolation (Rosic and Hoegh-Guldberg 2010) and cDNA synthesis were carried out for *Symbiodinium* cultures and from an *Acropora millepora* egg-sperm sample (free of algal symbionts).

### Synthesis of cDNA for qPCR

Reverse transcription was performed using QuantiTect® Reverse Transcription Procedure (Qiagen, Australia). Briefly, 0.5 μg of purified total RNA was used per reaction and incubated in gDNA Wipeout Buffer at 42°C for 2 min to eliminate traces of genomic DNA, followed by reverse transcription at the same temperature for 30 min. The obtained cDNA was used as a template in the qPCR analysis and diluted 1:10 prior to use.

### Primer design

Sequencing primers were designed to amplify the *Hb*-1 *and Hb*-2 sequences from *Symbiodinium* (C3) EST database as outlined in Table 2 using Primer Express® software v2.0 (Applied Biosystems, USA). The genes used for real-time RT-PCR analysis, GenBank accession numbers, and primers sequences are listed in Table 2. To confirm the reproducibility of the primers within *Symbiodinium* cultures and the absence of coral RNA, a standard PCR amplification was carried out using a selection of primers (Table S1) as previously described (Rosic et al. 2010, 2011b). PCR conditions were as follows: initial step at 94°C for 1 min, followed by 35 cycles of 94°C 20 sec, 60°C 20 sec, and 72°C 1.5 min, with a final extension phase at 72°C for 10 min, followed by samples storage at 4°C.

**Table 2:** GenBank accession numbers, gene name and primer sequences of putative *Hb* genes that were used for real-time RT-PCR analyses including the sequences of reference genes (*Tub*, *Rp-S4* and *SAM*) adopted from Rosic et al. (2011b)

Symbol	Gene Name	Forward Primer sequence (5′-3′)Reverse Primer sequence (5′-3′)	GeneBank Accession Number
*Hb-1*	*Hemoglobin-1*	CCGACGAGCCKTTGGATCCGCCACCTTCTTGAAAGTG	EH035884
*Hb-2*	*Hemoglobin-2*	TTGGTGCCCATGTTGCAAAGTATTCTGGCTTCAGGCCATATC	EH038142
*Tub*	*Beta-Tubulin*	TGACGCAGCAGATGTTTGATGCGACATACGTCCACGGAAGAG	EH037669
*Rp-S4*	*Ribosomal protein S4*	CCGCACAAACTGCGTGAGTCGCTGCATGACGATCATCTT	EH036413
*SAM*	*S-adenosyl-L-methionine synthetase*	GCCTACATTTGCCGACAGATGAATGGCTTGGCAACACCAAT	EH036622

### Quantitative PCR and gene expression analysis

The quantitative PCR assays were done by an Eppendorf 5075 (Applied Biosystems, USA) robot using SYBR Green PCR master mix (Applied Biosystems, Warrington, Cheshire, UK) in 384-well plates in a 7900HT Fast Real-time PCR System (Applied Biosystems, USA). PCR conditions were as follows: initial denaturation of 10 min at 95°C, followed by 45 cycles of 95°C for 15 sec and 60°C for 1 min. At the end, a dissociation step was included: 95°C for 2 min, 60°C for 15 sec, and 95°C for 15 sec. The final reaction volume was 10 μL and included 300 nmol/L of primers. All reactions were carried out in three technical replicates. The expression levels of targeted *Hb*-1 *and Hb*-2 genes were quantitated according to geNorm directions (Vandesompele et al. 2002). The relative quantitation method was applied for the relative abundance estimation of analyzed *Hb* genes using the best reference genes that showed the most stable expression patterns and specificity for *Symbiodinium* cultures (Table 1), as well as the absence of coral origin (Rosic et al. 2010, 2011a). The expression of each gene was determined from *C*_T_ (cycles threshold) value that corresponds to a number of cycles required for the PCR amplification to reach a fixed threshold in the exponential phase (Walker 2002). A specific threshold of 0.1 was used for obtaining *C*_T_ values that were transformed into quantities using maximal PCR efficiency for each gene (E = 2). The real-time dissociation curve was used to check for the presence of a unique PCR product. Following the normalization strategy outlined in Vandesompele et al. (2002) and using geNorm software (http://medgen.ugent.be/∼jvdesomp/genorm/), the stability of House Keeping Genes (HKGs) expression was tested during exposure to thermal and nutrient stress. The relative *Hb* genes quantities were normalized to the reference genes with the most stable expression pattern as defined by the geNorm analysis (Vandesompele et al. 2002) that are listed in Table 2.

### Statistical analysis

Statistical analyses were completed using the Statistica 9.0 software (Statsoft Inc., Tulsa, USA). All data were tested for normality and homogeneity of variance. Relative gene expression of the algal *Hb*-1 *and Hb*-2 in corals exposed to elevated temperatures and nutrient stress was compared with control corals at each time point using a *t-*test (*n* = 4). Values were considered significantly different if the *P* value was <0.05. Throughout the article, values are expressed as means ± standard deviations (SD).

## Results

### Molecular characterization of hemoglobin-like genes

In this study, we report two new *Hb*-like genes (*Hb*-1 and *Hb-*2) isolated from a *Symbiodinium* (C3) expressed sequence tags (ESTs) library (Leggat et al. 2007). Applying the additional sequencing of this library, we have recovered the full-length sequences of two hemoglobin genes including four isoforms (Fig. 2). Our results confirmed the algal origin of *Hb* sequences within the transcripts acquired from *Symbiodinium* cultures (Fig. 2B) and additional sequence polymorphism among different *Symbidinium* types (A2 and B2 types).

Despite considerable differences at both the C- and N- terminal, using blastp within the encoded Hb sequence, we discovered the presence of a conserved globin domain. Additionally, a highly conserved hemoglobin residue, the proximal histidine (F8), has been preserved in all protein sequences (Fig. 2). The sequence identity among Hb-1 isoforms was between 87% and 97%, whereas Hb-2 isoforms contained 78–99% of sequence identity. Between the two algal hemoglobin proteins, 15–21% sequence identity has been preserved within their primary structure. Within the sequences of *Hb*-1, we revealed an ORF of 522 bp, encoding 174 amino acids with a molecular mass of 18.9 kDa. Sequence polymorphisms among *Hb-*1 isoforms were noticed throughout the whole ORF, especially at the 3′ end. Due to redundancy of the genetic code, only five different amino acids were obtained at the protein level from 21 single nucleotide polymorphisms (SNPs) within *Hb*-1 isoforms. The second hemoglobin-like gene, *Hb-*2 contained also four isoforms, characterized by a substantial difference at the 5′ end and a different position of the ATG start codon. This resulted in the recovery of two ORFs (744 bp and 597 bp) for a putative *Hb*-2 gene. The deduced protein sequences of Hb-2 isoforms were 247 and 198 amino acids with molecular masses of 27 kDa and 21.8 kDa, respectively.

The best BLAST (blastx) hits (E < 1.0 × 10^−5^) of proposed *Symbiodinium Hb* genes are provided in Table 1. Conserved globin domains have been recovered in both predicted *Hb* genes using blastp option including the heme-binding site (Fig. 2). Neither transmembrane areas nor chloroplast transit peptides were detected in Hb sequences. The sequence analyses demonstrated the closest similarity of encoded Hb-1 and Hb-2 sequences to globin-like proteins such as hemoglobin from bacteria (*Sorangium cellulosum*), globin from brown algae (*Ectocarpus siliculosus*), putative nitric oxide dioxygenase from bacteria (*Bradyrhizobium* sp.), as well as to non-vascular plant hemoglobin in the case of Hb-1 or cytoglobin for Hb-2 (Table 1). Using additional Position-Specific Iterative BLAST (PSI-BLAST), the third iteration results included a number of hits to microbial flavohemoproteins indicating distant evolutionary relationship of *Symbiodinium* Hb proteins with this group of globin proteins that are characterized by the presence of a globin domain fused with a ferredoxin reductase-like FAD/NAD-binding domain and distinguished with a role in NO detoxification.

Predicted secondary structure indicated the presence of eight helices for Hb1 protein sequences, whereas seven helices and two strands were detected in Hb-2 (Fig. 3). The 3D protein structural predictions revealed the possible binding sites (BS), with a BS-score >1 that reflects a significant match and top prediction corresponding to Heme-proteins. For Hb-1, the top template protein with similar binding site is 1o1iA (Cyanomet hemoglobin) and for Hb-2 is 1cqxA (flavohemoglobin from *Alcaligenes eutrophus*). Predicted binding sites residues for heme (ligand) based on the 3D model of Hb-2 are as follows: Lys19, Pro31, Ser32, Asn33, Leu68, His72, Tyr75, Leu77, Tyr81, Phe82, Ala85, Ser113, Phe116, Met117, Met120 (absolutely conserved His residue is underlined) and indicated in Fig. 3C, D.

### Phylogenetic analysis of Hb-like genes

**Figure 4:**
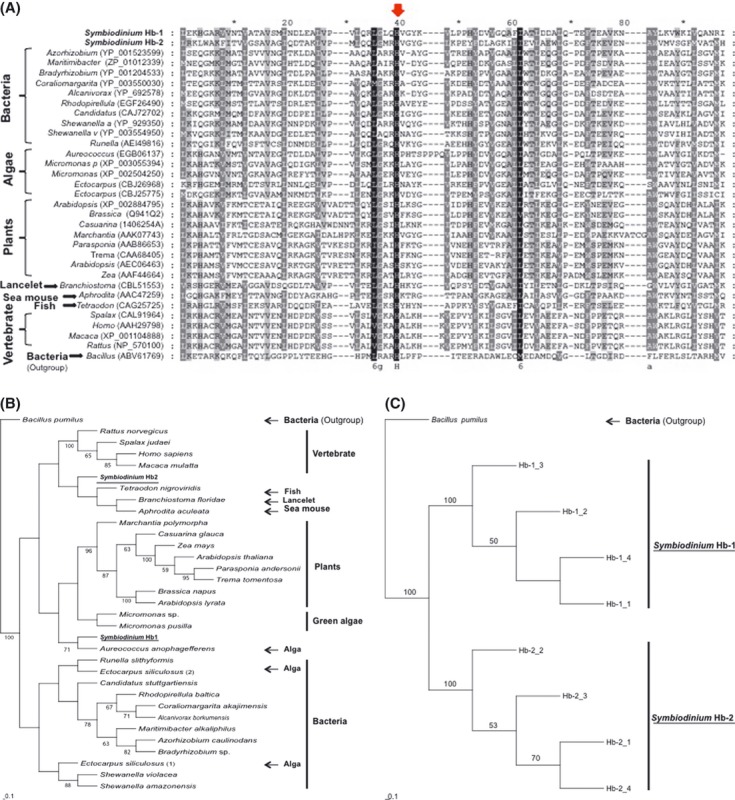
Multiple sequence alignment of the *Symbiodinium* encoding sequences and their homologs (A). Phylogenetic analyses of hemoglobin-like protein sequences from coral dinoflagellates and other organisms (B) including only Hb isoforms from *Symbiodinium* (C). The molecular phylogenetic tree of Hb-like homologs was based on the comparison of 90 amino acid residues of the heme-binding domain. Gene accession numbers (shown in the parentheses) were attained from the NCBI database (non-redundant protein sequences database). The phylogenetic tree was tested using a 500-replicated bootstrap analysis (Felsenstein 1989) and the results higher than 50% are indicated at each node. A distance method using maximum likelihood estimates was based on the Dayhoff PAM matrix (PHYLIP, Phylogeny Inference Package). The scale for the branch length (0.1 substitutions per site) is presented under the tree. Outgroup used is indicated on the figure.

For the construction of the phylogenetic trees, we used alignments of the deduced amino acid sequences of *Symbiodinium Hb*-like genes and the sequences of other globin proteins that showed the highest homology based on the BLAST search (Fig. 4A). The phylogenetic analyses identified the existence of two independent hemoglobin proteins from coral endosymbionts Hb-1 and Hb-2 (Fig. 4). An evolutionary relationship between *Symiodinium* Hb-1 and algal globins was shown after clustering with a good bootstrap value with their homolog from microalgae *Aureococcus anophagefferens*, which is one of Harmful Algal Bloom species. The encoding sequences of Hb-2 formed a group that clustered with their metazoan globin counterparts including fish, sea mouse, and ancient lancelet, although deficient of a strong bootstrapping.

### Expression of Hb-like genes under thermal and nutrient stress conditions

**Figure 5:**
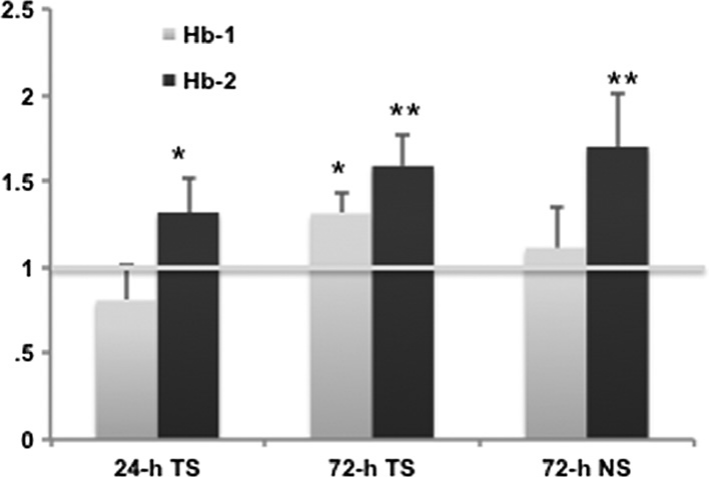
Relative quantitation of the *Hb* genes expression level after exposure to thermal stress (TS) for a 24-h period, a 72-h period, and Nutrient stress (NS). Data normalization was done using the most stable reference genes as defined by the geNorm analysis: *Rp-S4* and *SAM* with *M* value of 0.39 and pairwise variations for *V*2/3 below recommended cut-off of 0.15 (Vandesompele et al. 2002). The horizontal line corresponds to control. Results are given as the mean of four biological replicates ± SD. The statistical difference between means is indicated as * (*P* < 0.05) or ** (*P* < 0.01).

In *Symbiodinium* cultures (C1), the lack or low levels of *Hb* mRNA were measured even when the axenic algal cultures were exposed to elevated temperatures (26°C and 32°C for a 24-h period; data not shown). Our results showed the presence of identical sequences corresponding to primers of *Hb*1 gene for both C1 and C3, ruling out the possibility of poor primer binding as a reason of low/lack of *Hb*1 gene expression in cultures (Fig. S1). However, the abundant mRNA levels of both Hb genes were detected when in symbiosis within the coral host *Acropora aspera*. Therefore, the regulation of the *Hb* gene expression was tested in the samples of *A. aspera* exposed to different thermal and nutrient stress conditions (Fig. 5). Results of the relative expression of *Hb* genes have been provided at each time point as the ratio for treatment and control (T:CTRL) for *Hb*-1 and *Hb*-2. The average expression stability value (*M*) of HKGs *Beta-Tubulin* (Tub), *Ribosomal protein S4* (Rp-S4), and *S-adenosyl-L-methionine synthetase* (SAM) was 0.389 and with pairwise variations for *V*2/3 below a 0.15 cut-off value as recommended by Vandesompele et al. (2002). A 31% increase in *Hb*-1 transcript abundance (*P* < 0.05) was observed after 72 hours of thermal stress. The expression of *Hb*-2 gene was raised by both thermal stress conditions. First, the 1-day thermal stress resulted in a 32% increase (*P* < 0.05) in *Hb-*2 transcript level. The 3-day thermal stress condition resulted in a 59% increase in *Hb*-2 expression (*P* < 0.01). Nutrient stress also resulted in the increased expression of *Hb*-2 gene by 70% (*P* < 0.01) after a 3-day period, whlereas the transcript level of *Hb*-1 was not significantly changed (Fig. 5).

## Discussion and Conclusions

An important strategy to increase stress tolerance in plants and therefore the survival rate includes the contribution of hemoglobin proteins (Dordas 2009). These heme-containing proteins represent an ancient class of ubiquitous oxygen-binding proteins (Vinogradov et al. 2006) that after the extensive evolutionary pressure acquired a number of new features enabling them to adapt to extreme conditions and to preserve their functionality (Perutz 1983). Consequently, these proteins have been used for monitoring the adaptive changes in organisms exposed to variable external conditions (Andersen et al. 2009). Recent advances in sequencing technologies have resulted in the discovery of *Hb*-like sequences in many prokaryotic and eukaryotic microorganisms including bacteria, yeasts, algae, protozoa, and fungi and the presence of microbial globins such as truncated hemoglobins (trHb), globin-coupled sensors (GCSs), and flavohemoglobins (flavoHbs) (Bonamore and Boffi 2008). In the present study, we report the presence of two globin proteins in coral dinoflagellates, which are represented with several isoforms and a highly conserved hemoglobin residue, the proximal histidine (F8) (Fig. 2). The predicated 3D protein structure confirmed the globin fold for these dinoflagellate proteins and their preserved tertiary structure (Fig. 3). Likewise, many different hemo-proteins found in nature have similar tertiary structure (globin fold), as well as evolutionary origin, although showing a huge variability in their amino acid sequences (Royer et al. 2005). Our phylogenetic studies revealed the existence of two separate groups of hemoglobin proteins (Fig. 4). The BLAST search confirmed the Hb-like origin of these proteins and additionally using PSI-BLAST, their evolutionary link with microbial flavohemoproteins. Molecular phylogeny suggested a close evolutionary relationship between the *Symbiodinium* Hb-2 and its metazoan cytoglobin counterparts that act as a NO scavenger and play a role in oxidative stress response (Trent and Hargrove 2002) indicating a possibility of contamination or horizontal gene transfer. However, as Hb sequences were recovered also in cultures of different *Symbidinium* types (Fig. 2B), a possibility of contamination has been excluded. Furthermore, microbial origin of eukaryotic globins has been proposed as a result of horizontal gene transfer, which has occurred in the past, during endosymbotic events and lead to the first establishment of mitochondria and plastids such as chloroplasts in the eukaryotic cells (Hoogewijs et al. 2012). The complex evolutionary origin of dinoflagellate genes involved in the biosynthesis of mycosporine-like amino acids was also recently reported (Rosic and Dove 2011; Rosic 2012), as well as the occurrence of these microbial genes within the coral genome (Shinzato et al. 2011).

The *Hb* expression patterns can be affected by a number of factors including hypoxia, organogenesis, pathogen infection, and ontogenesis (see review by Kosmachevskaya and Topunov 2009). Our results revealed differential gene regulation of two algal Hbs proteins when the coral–dinoflagellate symbiosis was exposed to thermal and nutrient stress. The increased transcript abundance of *Hb-*1 after a 3-day period of thermal stress and even more inducible *Hb-*2 expression to both thermal and nutrient stress conditions (Fig. 5) may be due to a Hb role in the cell protection and in the process of scavenging NO (Dordas 2009; Kosmachevskaya and Topunov 2009). Consequently, it could be expected that *Symbiodinium* Hbs may also be involved in the metabolism of NO during thermal stress. The NO molecule is known as a very potent signaling molecule involved in a number of biological processes such as initiating host immunity response against pathogen invasion (Wang and Ruby 2011), as well as in signaling within coral–algal symbioses (Safavi-Hemami et al. 2010). A high level of NO production in the sea anemone exposed to elevated sea temperature can lead to the collapse of cnidarian–dinoflagellate symbiosis (Trapido-Rosenthal et al. 2001; Perez and Weis 2006). Additionally, nitrate, nitrite, and NO were found to induce the synthesis of non-symbiotic-Hb (Nsgb) in plants (Wang et al. 2000; Ohwaki et al. 2005), while Nsgl functioned as a NO dioxygenase, modulating NO metabolisms, and NO detoxification (Dordas et al. 2003b; Hebelstrup et al. 2007). Here, we also report a further increase in *Hb*-2 expression by 70% when coral–dinoflagellate symbiosis was exposed to ammonium-enriched seawater for a 3-day period. Nutrient over-enrichment is considered as one of the leading factors leading to coral decline (Szmant 2002). As algal endosymbionts and their invertebrate host exchange nutrients and metabolic products (Venn et al. 2008; Yellowlees et al. 2008), they also show the capacity to quickly fix nitrogen from the seawater enriched with ammonium, with much higher intake reported for symbiotic dinoflagellates compared with the host (Pernice et al. 2012). Nitrogen assimilation has been stimulated by over-expression of plant *Hbs* (class 1) that removes NO acting as an inhibitor of nitrogenase (Shimoda et al. 2009). Consequently, elevated transcript levels of *Hb*-2 mRNA reported here may indicate a potential role of this Hb form in NO detoxification and also enhancing the process of nitrogen assimilation in coral endosymbionts. Future studies are needed to determine the molecular mechanism of nitrogen absorption and NO detoxification and the role of Hb proteins during these processes.

Low *Hb* transcript levels observed for *Symbiodinium* cultures and the abundant and inducible expression of *Hb* genes *in hospite* may suggest that algal *Hb* gene expression requires the symbiotic condition or alternatively the Hb importance for symbiosis as seen in some plants (Bustos-Sanmamed et al. 2011). A lack or low transcript abundance for catalase, an antioxidant enzyme, has been also reported for *Symbiodinium* cultures (Bayer et al. 2012), whereas a high level of gene expression was detected in the cnidarian–dinoflagellate symbiosis (Sunagawa et al. 2009).

Despite the large ecological and socio-economic importance of coral reefs worldwide, our understanding of their ability to adjust to changing environmental conditions is poorly developed. A number of mechanisms have been proposed to potentially increase the coral–algal stress tolerance including inducible HSPs, production of oxidative enzymes, and fluorescent coral pigments (Coles and Brown 2003; Baird et al. 2009). The differences in stress tolerance found in corals could potentially be driven by genetic adaptation and/or phenotypic acclimatization (Weis 2010). Phenotypic plasticity, in response to thermal stress, has been reported for both partners in symbiosis (see review Weis 2010). Photo-acclimation of symbiotic dinoflagellates to high light levels can lead to higher thermal tolerance (Robison and Warner 2006). Previous exposure to temperature fluctuations in the environment can positively influence coral thermal tolerance (Oliver and Palumbi 2011). Hemo-proteins such as hemoglobins have been implicated as an important indicator of an organism's capacity to respond to environmental change due to their involvement in oxygen transport and related metabolic processes (Andersen et al. 2009). Our research suggests that *Symbiodinium Hb* genes, in particular *Hb*-2, play a role in the mechanisms of the early stress response during exposure of coral–dinoflagellate symbiosis to thermal and nutrient stress. Additional research is needed to elucidate the exact mechanisms of algal *Hb* transcriptional regulation upon exposure to stress and functional significance of hemoglobin polymorphism in *Symbiodinum*. In conclusion, this research provides new insights into the molecular changes occurring in symbiotic dinoflagellates under stress. Differential gene expression patterns of these highly polymorphic hemoglobin-like proteins of coral dinoflagellates indicate that these universal globin proteins may play an important role in the coral–algal stress response potentially through physiological acclimatization and/or evolutional adaptation to climate change.

## Supporting Information

**Figure S1**. Multiple sequence alignment of the Symbiodinium Hb-1 encoding sequences from C3 type (isoforms 1–4) and C1 type (Hb-1_C1) was done with Clustal W (A). Nucleotide and deduced amino acid sequences of Hb1 from Symbiodinium C1 type; the positions of primers (forward and reverse) used for qPCR analyses were indicated with arrows (B).

**Table S1**. A set of forward and reversed primers used for amplification of the full length of hemoglobin open reading frame (ORF).
