# Supplementary material for: New-old hemoglobin-like proteins of symbiotic dinoflagellates
Source: Ecol Evol. 2013 Feb 26;3(4):822–34. doi: 10.1002/ece3.498 (PMC3631397; doi:10.1002/ece3.498)
Supplement: Supplementary file 1 [file ece30003-0822-SD1.docx]

Supplementary Table 1. The list of primers used for obtaining a full-length of an ORF for two *Hb* genes.

| **Primer Location** | **Primer Name** | **Primer sequence (5’-3’)** |
| --- | --- | --- |
| pGEM vector | M13 Forward  M13 Reverse | GTAAAACGACGGCCAGTG  GGAAACAGCTATGACCATG |
| *Hb-1* | Contig1-Forward1  Contig1-Forward2  Contig1-Reverse1  Contig1-Reverse2 | ATGGCTGCTGGATCCCCTGTGAATG  AATGCCACCGAGCTTCCACTTGACTTG  GGCCTGCACAATCTTCCACACCTTCA  GGCCTGCACAATCTTCCACACC |
| *Hb-2* | Contig2-Forward1  Contig2-Forward2  Contig2-Reverse1  Contig2-Reverse2  Contig2-Reverse3  Contig2-Reverse4 | TTTCACCCAGCCAGTCCAACCTAACAGC  CGCTACAAGGACTGGGATACTGATGAGG  CGAAGTGCCGGGGAATTGGAAGGA  CCCCCAGCCACTCCGCCAGAAC  GCAACCATGAAGCCAGACACCA  CCCCAGCCACTCCGCCAGAAC |
