# Supplementary figures and images for: New-old hemoglobin-like proteins of symbiotic dinoflagellates
Source: Ecol Evol. 2013 Feb 26;3(4):822–34. doi: 10.1002/ece3.498 (PMC3631397; doi:10.1002/ece3.498)

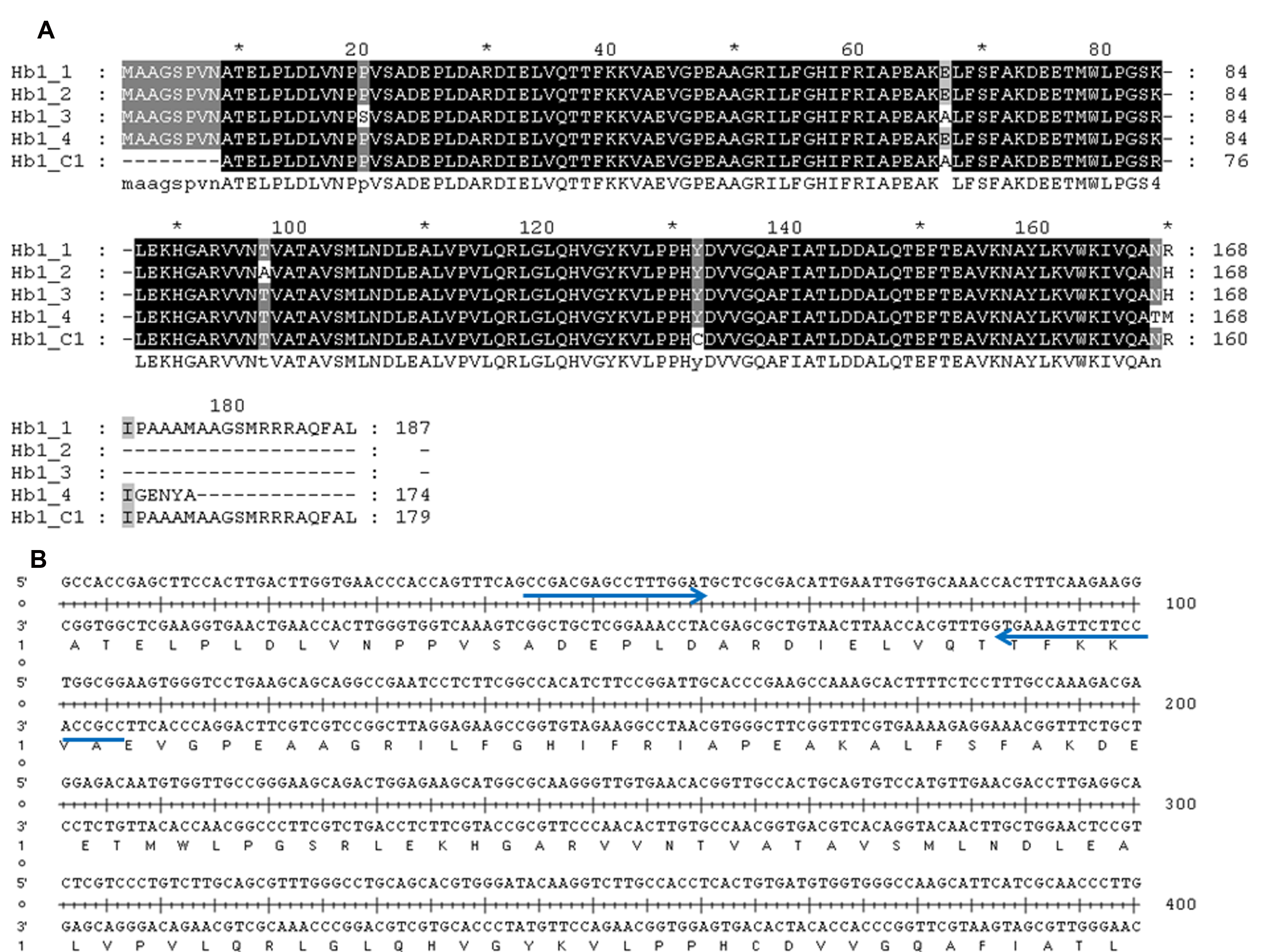

Supplement: Supplementary file 2 [file ece30003-0822-SD2.tif]
